# Embodiment as outcome: the translational test for soft robotics

**DOI:** 10.1016/j.ebiom.2025.106031

**Published:** 2025-11-12

**Authors:** eBioMedicine

Agency and adoption now define success in assistive wearables. Soft robotics represent an emerging approach in assistive and rehabilitation technologies, distinguished by their use of materials that closely mimic biological tissues. This technology is being used in both home and clinical therapeutic settings to support active strengthening, range of motion exercises, and assistance with activities of daily living, among other applications. Unlike traditional rigid robots designed for precise, high-force movements, soft robots prioritise safety, comfort, and usability, making them suitable for neurorehabilitation and assistance across a broader patient spectrum. Although still in early stages, ongoing advances in manufacturing and materials offer cautious optimism that these devices will become more affordable and accessible.

The benchmark for success is shifting: raw torque is no longer enough. The new standard is agency—ie, the ability to help without hindering. The discussion of embodiment—a user's sense of ownership and control—has moved from philosophy to protocol. An August, 2025, *Nature Communications* study by Arnold and colleagues introduced a personalised controller that learned an individual's movement signatures in minutes and ran on-device, in real time. By combining inertial and soft compression sensors with a physics-based model that captures how movement patterns depend on past actions, the system provided support during upward motion and relaxed when lowering—not restricting the users' movement when unpowered. Across participants with stroke and amyotrophic lateral sclerosis (ALS), the controller improved whole-arm movement quality: larger range of motion at the shoulder, elbow, and wrist; less trunk compensation (ie, use of the torso to assist arm or hand motion when upper limb mobility is limited); and cleaner hand paths (ie, more efficient movement of the hand when reaching from one point to another). The system also reduced the amount of support or resistance the robot applies to assist the arm's downward movement by nearly one-third compared with a baseline controller, allowing for more natural and comfortable motion. Most importantly, the gains were shown not just in the lab but at home, across days and tasks.

Evidence from other groups points to the same translational progress, assistive devices improving daily function and remaining useable beyond the laboratory. In 2023, Proietti and colleagues tested a portable textile shoulder support in people with ALS. The device restored useful elevation and daily functions without training, added approximately 150 g per arm, and was imperceptible to the user when unpowered. Two participants were followed for more than 6 months as motor function declined, showing the system could adapt over time with a 30 s automatic calibration. A short automatic calibration is a reminder that usability, not just accuracy, decides whether a device is worn after the first week.

The technical foundations that could make embodiment measurable have already been outlined: combining signals from multiple sensors, adapting control with the user in the loop, embedding flexible electronics, and developing biomechatronic chips optimised for wearable use. Arnold and colleagues implemented elements of this roadmap, integrating different kinds of inputs, adapting quickly to individual users, and designing systems that do not restrict range of motion when inactive.

Devices are now judged as much by comfort, transparency, and reliability as by force output. Other findings emphasise this same convergence. Cloud-linked electromyograph systems can classify four upper-limb intentions with almost 96% accuracy at 500–550 ms latency while driving soft pneumatics. This finding proves capability, but also the need to test whether the model can quickly and smoothly process new sensor data on the device without slowing down the controller. A textile exomuscle such as Myoshirt can increase endurance by 36% and reduce muscle activation by half, meaning the users needed only half as much effort to complete the same task. For two users of this textile exomuscle who had motor impairments, endurance rose sharply and range of motion stayed within daily-life requirements.

Several practical questions emerge: who continues to wear these devices a month after fitting, and why? When does assistance feel supportive, and when does it undermine agency? How much calibration or setup is acceptable to users, and how responsive should the real-time feedback loop (whereby the robot adjusts assistance based on user movement) be for everyday use?

To get relevant answers to these questions, future work should aim to recruit across diagnoses and prespecified subgroups; heterogeneity is the rule, not the exception. Researchers should investigate not just how people move with these devices, but how well they move, how easy it is for them, and how they feel about using the device. These measures should be included in an embodiment index so torque is not mistaken for value. Studies should aim to keep track of how quickly and smoothly the device responds, how setup and adjustments are done, and how often people actually use the device at home. Fatigue should also be measured and reported: sometimes the benefit is not a new motion but another hour before the arm is too tired to move.

Safety, equity, and cost matter as much as performance. It is important to define acceptable parameters for muscle activity, joint loading, and pain to prevent over-reliance or harm; to test across body types, skin tones, ages, and socioeconomic groups; to report repairability and total cost of ownership; to clarify who calibrates and maintains the system, and how much training time is required; and to include a clear measure of time to independent use. These factors decide whether a device ends up integrated into daily life or abandoned in the drawer.

Embodiment might ultimately be less a metric than a relationship. Trust is accumulated over small, repeated successes—a more accessible shelf, a steadier pour, or a task finished before fatigue intrudes. The science is ready; multimodal sensing, unrestrictive control, and flexible interfaces now exist. The next step is to test these systems at home, with diverse users, and with clear reporting. If people get more done with less effort and keep the device on, we will know the field is moving in the right direction. Embodiment, not perfection, is progress.

